# Visual assessment of interactions among resuscitation activity factors in out-of-hospital cardiopulmonary arrest using a machine learning model

**DOI:** 10.1371/journal.pone.0273787

**Published:** 2022-09-06

**Authors:** Yasuyuki Kawai, Hirozumi Okuda, Arisa Kinoshita, Koji Yamamoto, Keita Miyazaki, Keisuke Takano, Hideki Asai, Yasuyuki Urisono, Hidetada Fukushima

**Affiliations:** Department of Emergency and Critical Care Medicine, Nara Medical University, Kashihara City, Nara, Japan; Fondazione IRCCS Policlinico San Matteo, ITALY

## Abstract

**Aim:**

The evaluation of the effects of resuscitation activity factors on the outcome of out-of-hospital cardiopulmonary arrest (OHCA) requires consideration of the interactions among these factors. To improve OHCA success rates, this study assessed the prognostic interactions resulting from simultaneously modifying two prehospital factors using a trained machine learning model.

**Methods:**

We enrolled 8274 OHCA patients resuscitated by emergency medical services (EMS) in Nara prefecture, Japan, with a unified activity protocol between January 2010 and December 2018; patients younger than 18 and those with noncardiogenic cardiopulmonary arrest were excluded. Next, a three-layer neural network model was constructed to predict the cerebral performance category score of 1 or 2 at one month based on 24 features of prehospital EMS activity. Using this model, we evaluated the prognostic impact of continuously and simultaneously varying the transport time and the defibrillation or drug-administration time in the test data based on heatmaps.

**Results:**

The average class sensitivity of the prognostic model was more than 0.86, with a full area under the receiver operating characteristics curve of 0.94 (95% confidence interval of 0.92–0.96). By adjusting the two time factors simultaneously, a nonlinear interaction was obtained between the two adjustments, instead of a linear prediction of the outcome.

**Conclusion:**

Modifications to the parameters using a machine-learning-based prognostic model indicated an interaction among the prognostic factors. These findings could be used to evaluate which factors should be prioritized to reduce time in the trained region of machine learning in order to improve EMS activities.

## Introduction

Low resuscitation success rates in out-of-hospital cardiopulmonary arrest (OHCA) events are a common problem encountered globally. To facilitate the development of outcome-improvement strategies, prehospital records in the standardized Utstein style have been collected by institutions globally for several decades [1]. Moreover, prognostic factors have been investigated using big data analysis [2, 3]. Several randomized controlled trials have also been undertaken to improve OHCA outcomes [4–7].

However, background heterogeneity cannot be ignored in large-cohort studies because of the heterogeneity in healthcare resources and registries [8, 9]. Additionally, including various cohorts in a dataset obscures the effect of interventions on subgroups [10]. Therefore, the improvement strategies identified in previous studies may be ineffective in some regions. Furthermore, because the evaluation of simultaneous interactions between multiple prognostic factors is difficult using conventional statistical approaches, previously reported approaches were focused exclusively on the effects of individual factors [11]. Consequently, exploring strategies for improving prehospital activities while prioritizing the provision of emergency medical services (EMS) has become difficult.

Recently, machine learning models have been utilized for prediction in OHCA, among which neural networks are reportedly the most accurate [11–17]. However, since interactions among the factors affecting the outcome are included in the structure of the neural network, changing a single factor may simulate situations that would be difficult to handle in a randomized controlled trial [12, 18].

In this study, we hypothesized that the effect of modifying the two factors of activity duration on prognosis would not be linear because of the interactions between the time components of EMS activity. Using a prognostic model constructed from EMS activity records according to a unified protocol, we evaluated the effects of simultaneous changes in the two EMS activity factors using visual representations. The model proposed in this study was developed using specific regional data. However, applying the underlying concepts with external data from diverse backgrounds could facilitate assessment of the current status of EMS activities in other regions and the suggestion of targets for the improvement of EMS activities specific to other regions.

## Materials and methods

### Study design

We conducted a retrospective analysis of the EMS activity in Nara prefecture, Japan as recorded in the prospectively collected Utstein style. This observational study was approved by the ethical review board of Nara Medical University (no. 2973). Further, as only anonymized EMS activity records were used, the requirements for informed consent were waived by the reviewing authority.

### Study population and data sources

Nara prefecture is a rural region with a population of approximately 1.4 million, 30.9% of which are 65 years and older [19]. Approximately 1000 OHCA cases occur in the prefecture annually, and the emergency team is dispatched to all cases in response to emergency requests. The EMS activities are performed following a unified protocol that conforms with the resuscitation guidelines and is reviewed every five years. The EMS activity records are subject to triple activity checks, and feedback are collected for each incident and compiled annually. This study examined all OHCA prehospital records where resuscitation was performed by the EMS in Nara prefecture between January 2010 and December 2018. Patients under 18 years and patients with non-cardiogenic cardiopulmonary arrest were excluded to reduce the variance in patient backgrounds.

### Local emergency medical system in the study area

The emergency medical service is activated and dispatched by the communication command center via an emergency call. Concurrently, the communication command center provides verbal CPR instructions for all suspected cases of cardiopulmonary arrest. A physician may accompany EMS at the request of the communications command center. The EMS service in this country can provide defibrillation for cardiopulmonary arrest patients. They are also capable of advanced airway management and adrenaline administration under the instruction of a physician. All cases are transferred to a hospital unless there are clear signs of death.

### Data collection and preprocessing

Twenty-four data items obtained from the prehospital Utstein-style EMS activity records were included in the study. The collected factors were preprocessed as follows. Categorical data were one-hot encoded, and missing data were encoded as missing data. (S1 Table) Continuous variables (age, number of defibrillations, and number of drugs administered) had no missing values and were standardized. However, certain variables that were used later for adjustment (time from call to contact, time from contact to arrival, etc.) were retained as continuous variables. Interventions that were not performed on all patients (e.g., defibrillation, drug administration) were one-hot-encoded for non-performance and for time from contact to performance. The preprocessing created 145 input factors. The details of the preprocessing are shown in S2 Table.

The machine learning model was constructed in this study with the aim of obtaining the neurological prognostic cerebral performance category (CPC) scores [20] recorded one month after the occurrence of cardiac arrest. The CPC score indicates the following patient categories: (1) mild or no neurological deficits, (2) moderate neurological deficits, (3) severe neurological deficits, (4) in persistent coma or vegetative state, and (5) deceased. The outcome was based on a binary classification (Yes/No) with a CPC1 or 2 (CPC1/2) as the output factor, indicating a favorable neurological outcome.

### Dataset usage and predictive model construction methods

**Fig 1:**
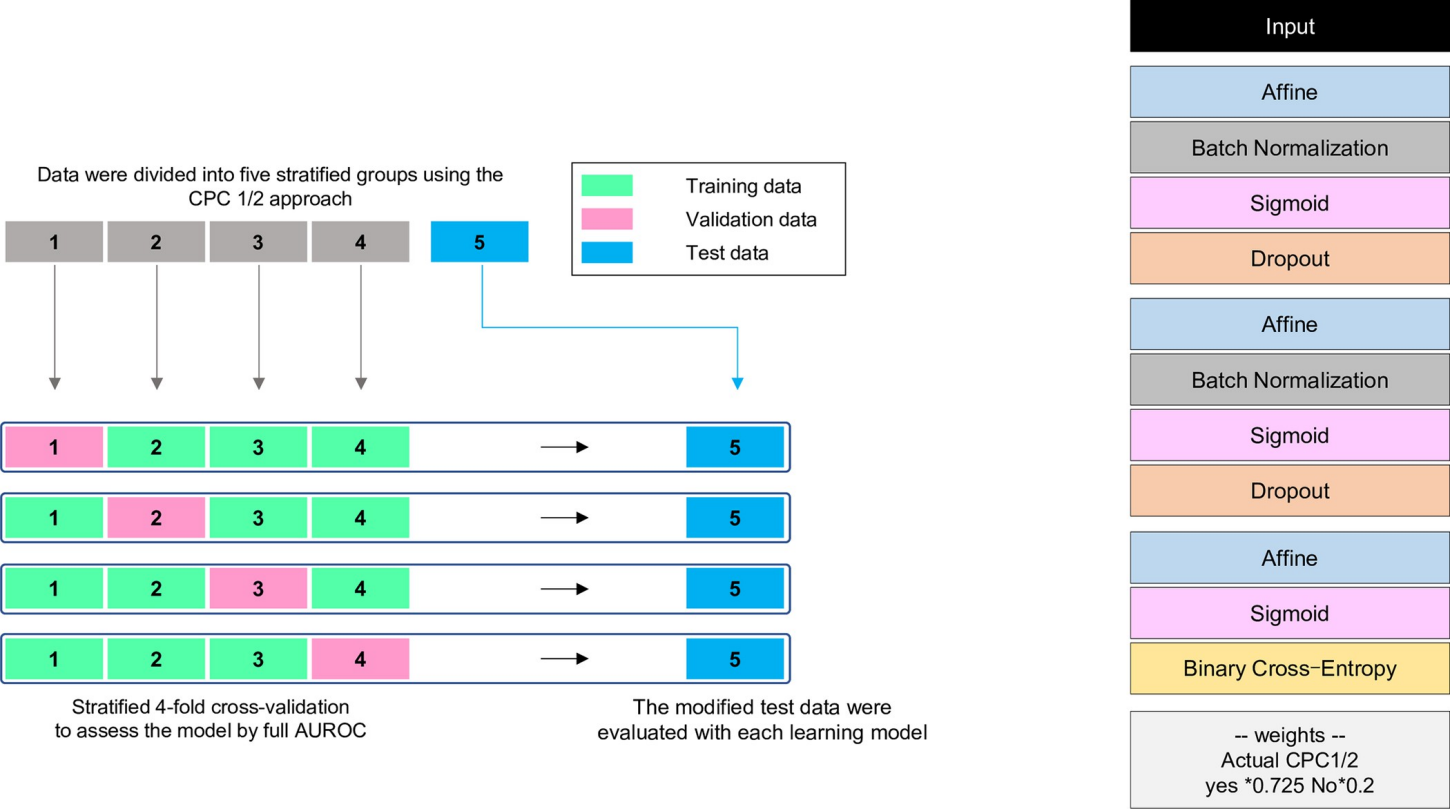
Overview of the data partitioning and stratified cross-validation method and neural network-based machine learning model. The model was developed using the stratified cross-validation method with CPC1/2. The machine learning model comprised a three-layer neural network, along with class weighting to improve the predictive precision for imbalanced data. CPC, cerebral performance category; BN, batch normalization; AUROC, area under the receiver operating characteristics curve.

The collected dataset was stratified using the CPC1/2 method and divided into five groups (Fig 1). One group was pre-separated from the other four groups used for training to be used as test data and modified data for the final constructed predictive models. The remaining data groups were used to train and validate four models via the CPC1/2 stratified cross-validation method.

The prediction model was built using a neural network that had the best average class sensitivity after several machine learning model trials. The compared methods included logistic regression, k-nearest neighbors, support vector machine, decision tree, random forest, XGBoost, and LightGBM. Increasing the layers improved the performance, but no accuracy improvement resulted above four layers. Consequently, after considering the training cost, we chose three layers. To reduce overfitting, Batch Normalization and Dropout were introduced into each layer. The Sigmoid function and Binary Cross-Entropy were used for the activation and loss functions, respectively. The network structure was optimized using the *Structure*.*Search* function available within the Neural Network Console (version 1.9.7587.58782, Sony; Tokyo, Japan). This function automatically optimizes the network structure to obtain a more accurate and computationally less-expensive alternative.

To evaluate predictions from imbalanced datasets, accuracy and area under the receiver operating characteristics curve (AUROC) are inadequate for predicting the minority class predictions individually [21]. Moreover, if the sensitivity of the majority class is inadequate, the predicted minority class (such as CPC1/2) can be enormous. Whereas previous studies often evaluated model accuracy using single class sensitivity [11, 12] or AUROC [13–17], the model in this study was created to accurately predict the number of minority class predictions. Class weighting for the loss function can effectively improve the sensitivity of the minority class. However, the sensitivity of the larger non-CPC1/2 class should be maximal because it significantly impacts the predicted CPC1/2 class. Nevertheless, class weighting involves a trade-off between the sensitivities of the CPC1/2 and non-CPC1/2 classes; therefore, balanced weighting must be considered to ensure the sensitivity of the CPC1/2 class does not decrease excessively. Consequently, in our model, the sensitivity of CPC1/2 (minority class) was set to approximately 80% to ensure the accurate prediction of the CPC1/2 sensitivity and that the value of the predicted CPC1/2 is controlled; the weights were adjusted to ensure that the sensitivity of not-CPC1/2 was the highest. Model construction, training, and validation were performed using Neural Network Console version 1.9.7587.58782 (Sony, Tokyo, Japan). The models were trained considering a batch size of 100 with 50 epochs and the Adam optimizer (learning rate = 0.001). All statistical analyses were performed using R 4.0.2 (R Development Core Team, Vienna, Austria). Differences were considered statistically significant at *p* < 0.05.

### Sensitivity analysis

To assess model validity, we performed a sensitivity analysis by modifying the adjustable prognostic factors. The previously reported prognostic factors include initial waveform rhythm, age, collapse to basic-life-support duration, arrival time after EMS dispatch, arrival time at hospital, and incident location [11, 22, 23]. For sensitivity analysis, we evaluated three of the adjustable factors: age and the times from call to contact and contact to arrival. Each parameter was added or subtracted independently, and the change in the predicted CPC1/2 was confirmed (i.e., age ± 5 years; time from call to contact +10 min and +20 min; time from contact to arrival, −5 min, +10 min, and +20 min).

### Simulation method with parameter manipulation

Using the constructed prediction model, we simulated the change in the predicted CPC1/2 by adding or subtracting time variables to the test data (n = 1654), previously separated from the training set beforehand. Time from arrival to defibrillation [24] and time to drug administration [25–27] have been reported as important prognostic factors of EMS activity time. Furthermore, defibrillation is a primary priority in all cases of shock-adapted waveforms. Therefore, the adjusted time factors included the elapsed time between (a) arrival of the EMS and arrival at the hospital, (b) arrival and first defibrillation, and (c) arrival and first-drug administration. Of these time factors, we adjusted (a) and (b), and (a) and (c) simultaneously. However, the time of the first defibrillation was adjusted for the 156 patients whose initial waveform was shockable. The initial drug administration time was adjusted for 588 patients, excluding those whose cardiac arrest was witnessed by firefighters, paramedics, or emergency lifesaver. Each of the four prediction models was evaluated against these adjusted test data to determine the average predicted CPC1/2. A heatmap was created for each result as a percentage increase/decrease from the average predicted CPC1/2 for the unadjusted test data (S1 Fig).

Continuous variables were described by their median and interquartile range, and categorical variables were expressed as percentages. Comparisons between the five divided groups were performed using the Kruskal–Wallis test for continuous variables and Fisher’s exact test for categorical variables. *p* value < 0.05 indicated a statistically significant difference.

## Results

**Fig 2:**
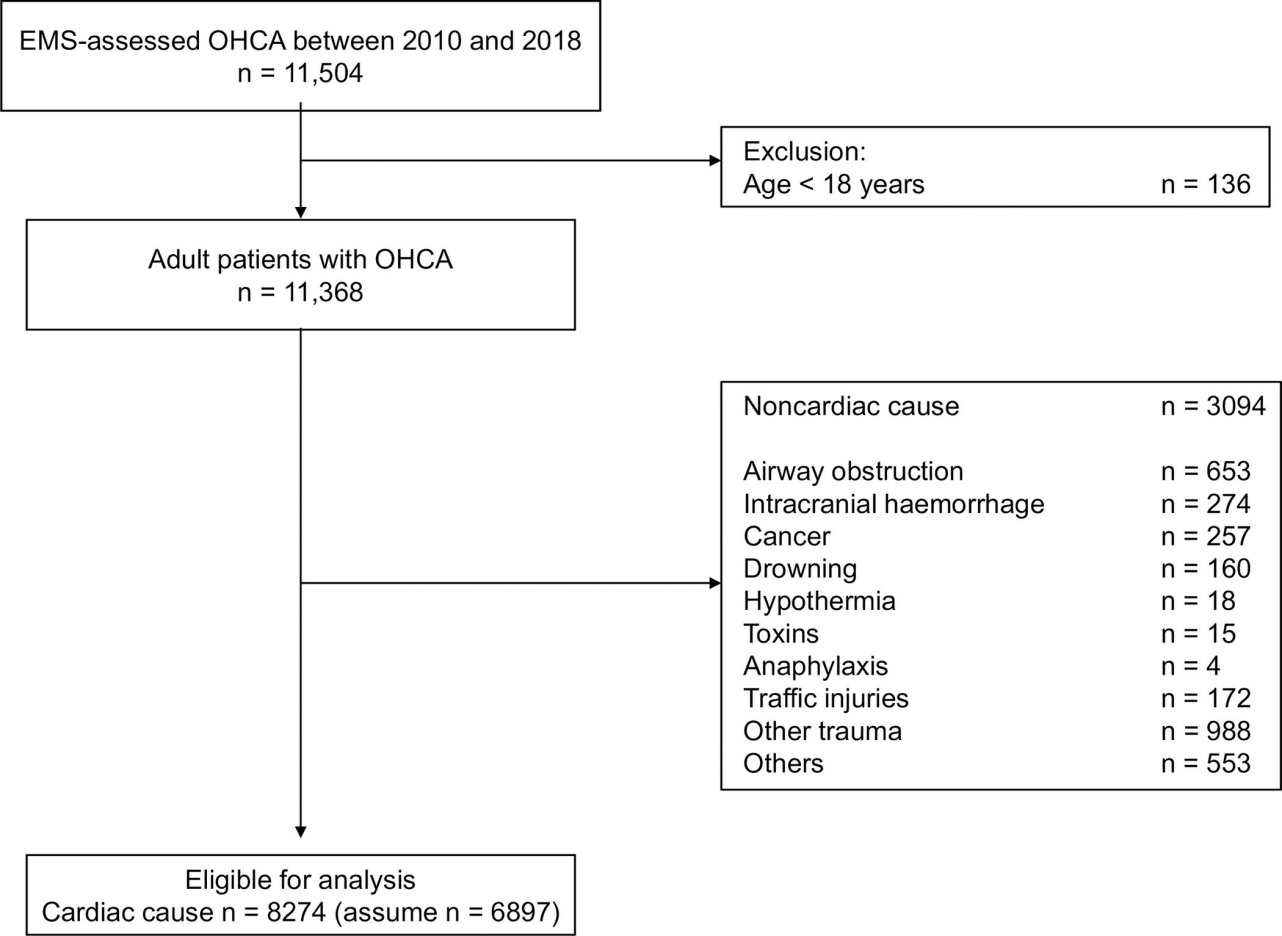
Patient selection flowchart. EMS, emergency medical services; OHCA, out-of-hospital cardiopulmonary arrest.

During the study period, 11,504 OHCA with cardiopulmonary resuscitation were recorded. Of these, 8274 patients (72%) met the inclusion criteria (Fig 2). Table 1 shows the background of the patients and the dataset created by dividing the patients into five stratified groups based on CPC1/2.

**Table 1:** Overall patient background and comparison between five groups classified by CPC1/2.

			Five groups (classified by CPC1/2)					
Variables		Overall	1	2	3	4	5	p value
		n = 8274	n = 1654	n = 1656	n = 1655	n = 1655	n = 1654	
Age (years), median [IQR]		81 [71, 87]	80 [72, 87]	81 [71, 87]	81 [71, 88]	80 [71, 88]	81 [71, 87]	0.98
Sex (male), n (%)		4592 (56)	907 (55)	912 (55)	909 (55)	942 (57)	922 (56)	0.73
Guideline2010, n (%)		4360 (53)	901 (55)	857 (52)	863 (52)	888 (54)	851 (52)	0.34
Guideline2015, n (%)		3914 (47)	753 (46)	799 (48)	792 (48)	767 (46)	803 (49)	0.34
Witnessed arrest, n (%)		3298 (40)	640 (39)	675 (41)	673 (41)	661 (40)	649 (39)	0.7
Type of witness, n (%)	Family	1692 (20)	318 (19)	347 (21)	361 (22)	326 (20)	340 (21)	0.77
	Friends	91 (1)	22 (1)	21 (1)	14 (1)	16 (1)	18 (1)	
	Colleagues	71 (1)	10 (1)	12 (1)	18 (1)	13 (1)	18 (1)	
	Passers-by	65 (1)	10 (1)	14 (1)	13 (1)	10 (1)	18 (1)	
	Other	703 (9)	151 (9)	139 (8)	132 (8)	151 (9)	130 (8)	
	Firefighter	2 (0)	1 (0)	0 (0)	1 (0)	0 (0)	0 (0)	
	Paramedic	172 (2)	25 (2)	40 (2)	33 (2)	37 (2)	37 (2)	
	Emergency lifesaver	502 (6)	103 (6)	102 (6)	101 (6)	108 (7)	88 (5)	
Bystander CPR, n (%)		4264 (52)	878 (53)	830 (50)	820 (50)	879 (53)	857 (52)	0.12
Bystander CPR (actions), n (%)	Compressions only	4247 (51)	876 (53)	824 (50)	817 (49)	878 (53)	852 (52)	0.1
	Compressions and ventilations	531 (6)	96 (6)	117 (7)	111 (7)	110 (7)	97 (6)	0.49
AED use by bystander, n (%)		134 (2)	24 (2)	32 (2)	22 (1)	30 (2)	26 (2)	0.62
EMS with emergency lifesaver, n (%)		8089 (98)	1622 (98)	1622 (98)	1619 (98)	1617 (98)	1609 (97)	0.6
EMS with medical doctor, n (%)		465 (6)	88 (5.3)	78 (5)	97 (6)	101 (6)	101 (6)	0.34
First monitored rhythm, n (%)	VF	704 (9)	134 (8)	137 (8)	149 (9)	138 (8)	146 (9)	0.95
	Pulseless VT	52 (2)	9 (1)	11 (1)	11 (1)	11 (1)	10 (1)	
	PEA	1669 (20)	342 (22)	330 (20)	349 (21)	306 (19)	342 (21)	
	Asystole	5416 (66)	1091 (66)	1090 (66)	1064 (64)	1105 (67)	1066 (64)	
	Other	433 (5)	78 (5)	88 (5)	82 (5.0)	95 (6)	90 (5)	
Defibrillation, n (%)		1019 (12)	197 (12)	200 (12)	217 (13.1)	200 (12)	205 (12)	0.84
Median time from contact with patients by EMS to shock by EMS, minute, median [IQR]		4 [3, 12]	5 [3, 12]	4 [3, 12]	5 [3, 12]	4 [3, 11]	4 [3, 12]	0.59
Frequency of prehospital defibrillation, median [IQR]		0 [0, 0]	0 [0, 0]	0 [0, 0]	0 [0, 0]	0 [0, 0]	0 [0, 0]	0.79
Routes of medication administration, n (%)		3778 (46)	721 (44)	785 (47)	738 (45)	752 (45)	782 (47)	0.12
Adrenaline, n (%)		2947 (36)	556 (34)	591 (36)	575 (35)	614 (37)	611 (37)	0.18
Median time from contact with patients by EMS to adrenaline (minute) median [IQR]		16 [12, 21]	15 [12, 21]	16 [12, 21]	16 [12, 21]	16 [12, 22]	16 [12, 22]	0.16
Frequency of prehospital adrenaline, n (%)		0 [0, 1]	0 [0, 1]	0 [0, 1]	0 [0, 1]	0 [0, 1]	0 [0, 1]	0.28
Advanced airway management, n (%)		7320 (89)	1475 (89)	1463 (88)	1459 (88)	1464 (89)	1459 (88)	0.89
Median time from call to contact with patients by EMS, minute, median [IQR]		8 [7, 10]	8 [7, 10]	8 [7, 10]	8 [7, 10]	8 [7, 10]	8 [7, 10]	0.74
Time from contact by EMS to hospital arrival (minute), median [IQR]		28 [22, 36]	28 [22, 36]	28 [22, 35]	28 [23, 36]	28 [22, 36]	28 [23, 36]	0.54
ROSC during transport, n (%)		824 (10)	175 (11)	148 (9)	170 (10)	167 (10)	164 (10)	0.58
CPC 1 or 2, n (%)		286 (4)	57 (3)	58 (4)	57 (3)	57 (3)	57 (3)	1

CPR: cardio pulmonary arrest; AED: automated external defibrillator; EMS: emergency medical services; VF: ventricular fibrillation; VT: ventricular tachycardia; PEA: pulseless electrical activity; IQR: interquartile range; ROSC: return of spontaneous circulation; CPC: cerebral performance category

**Fig 3:**
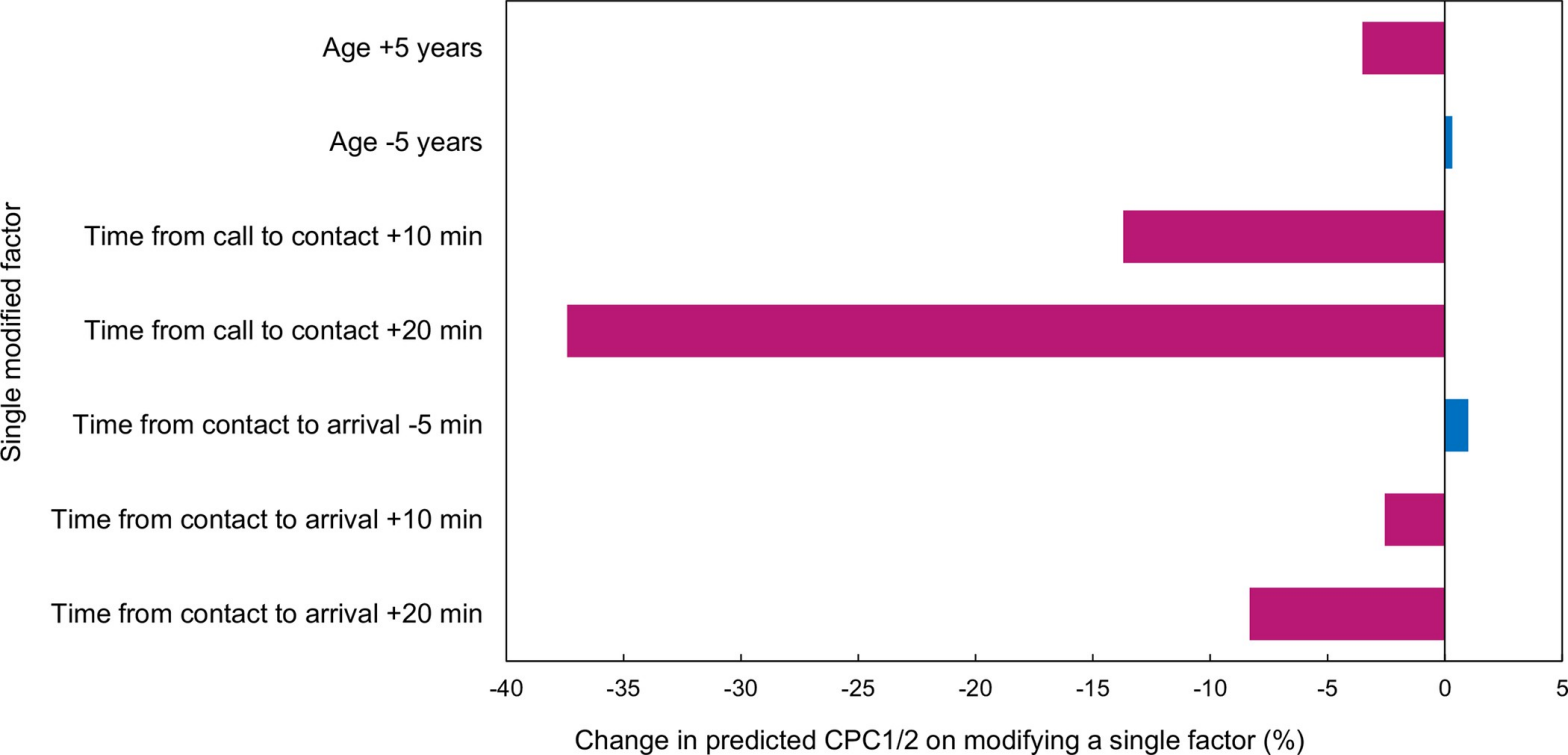
Modification of single factors changes the predicted CPC1/2. Changes in predicted outcome obtained by modifying a single factor. A higher age worsened the prognosis, and vice versa. Time delay worsened the prognosis, whereas reducing the duration improved the prognosis. CPC, cerebral performance category.

Fig 1 depicts the prediction model used in this study. The number of multiplications and additions in a single training run of the neural network model constructed in this study was approximately 44,000. The average class sensitivities of the four cross-validation models were 0.87, 0.86, 0.91, and 0.90, and the full AUROC was 0.96 (95% CI 0.94–0.97). Using this model to validate the test data, the mean class sensitivities were 0.86, 0.88, 0.87, and 0.88, and the full AUROC was 0.94 (95% CI 0.92–0.96) (S2 Fig). Fig 3 shows the sensitivity analysis results. The influence of increasing/decreasing age and reducing/extending time on the outcome was confirmed to exhibit the same trend as the influence of the previously reported prognostic factors [11].

**Fig 4:**
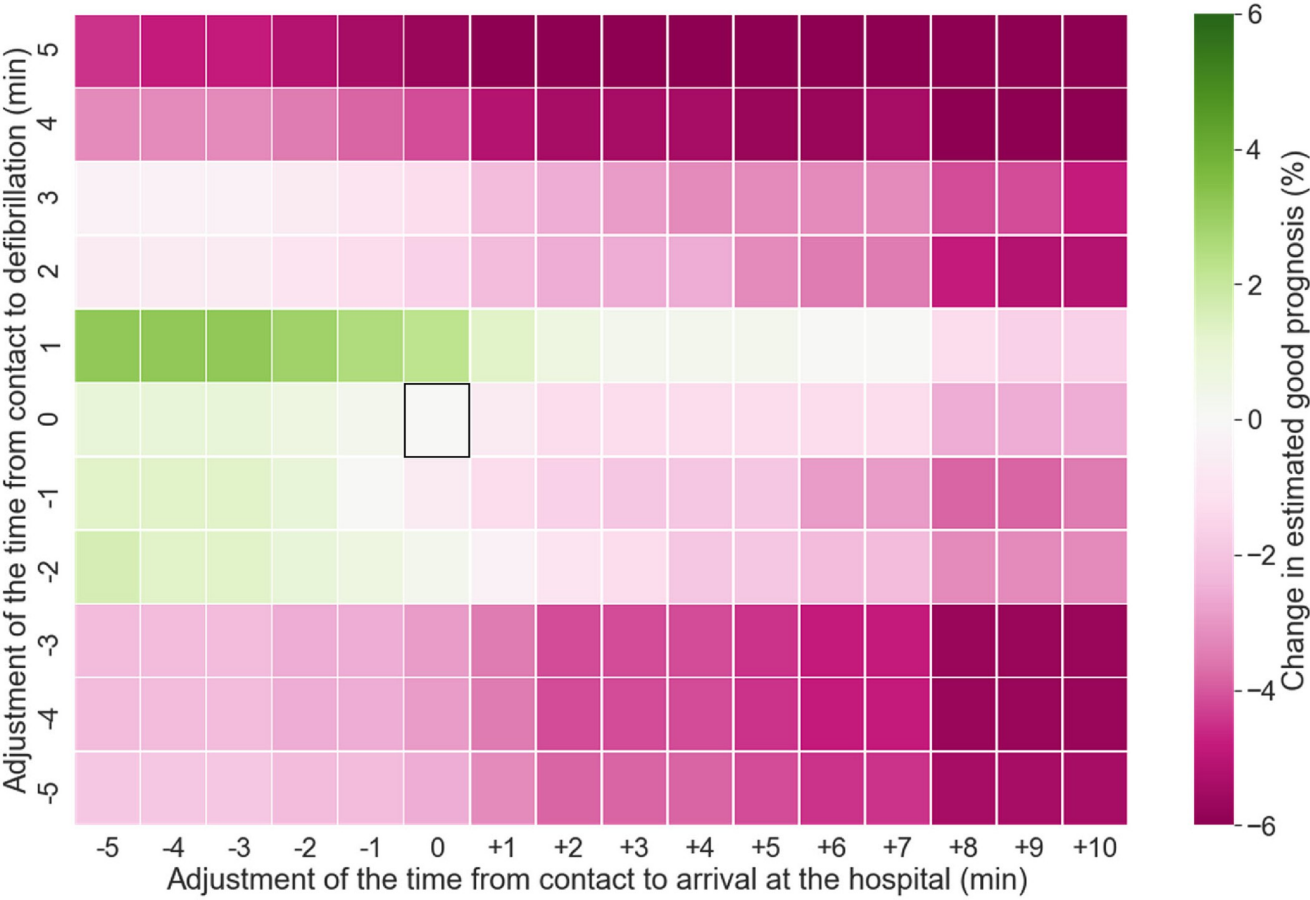
Modification of time from ambulance contact to hospital arrival and first defibrillation. The square box indicates no adjustments. The two ends of the color spectrum show a 6% increase and decrease in predicted CPC1/2, respectively. Overall, reducing the time for hospital arrival improved outcomes, whereas delays worsened them. A reduced time to defibrillation strengthened this trend, but it was obscured when the time to defibrillation was reduced by more than 3 min or delayed by more than 2 min. CPC, cerebral performance category.

**Fig 5:**
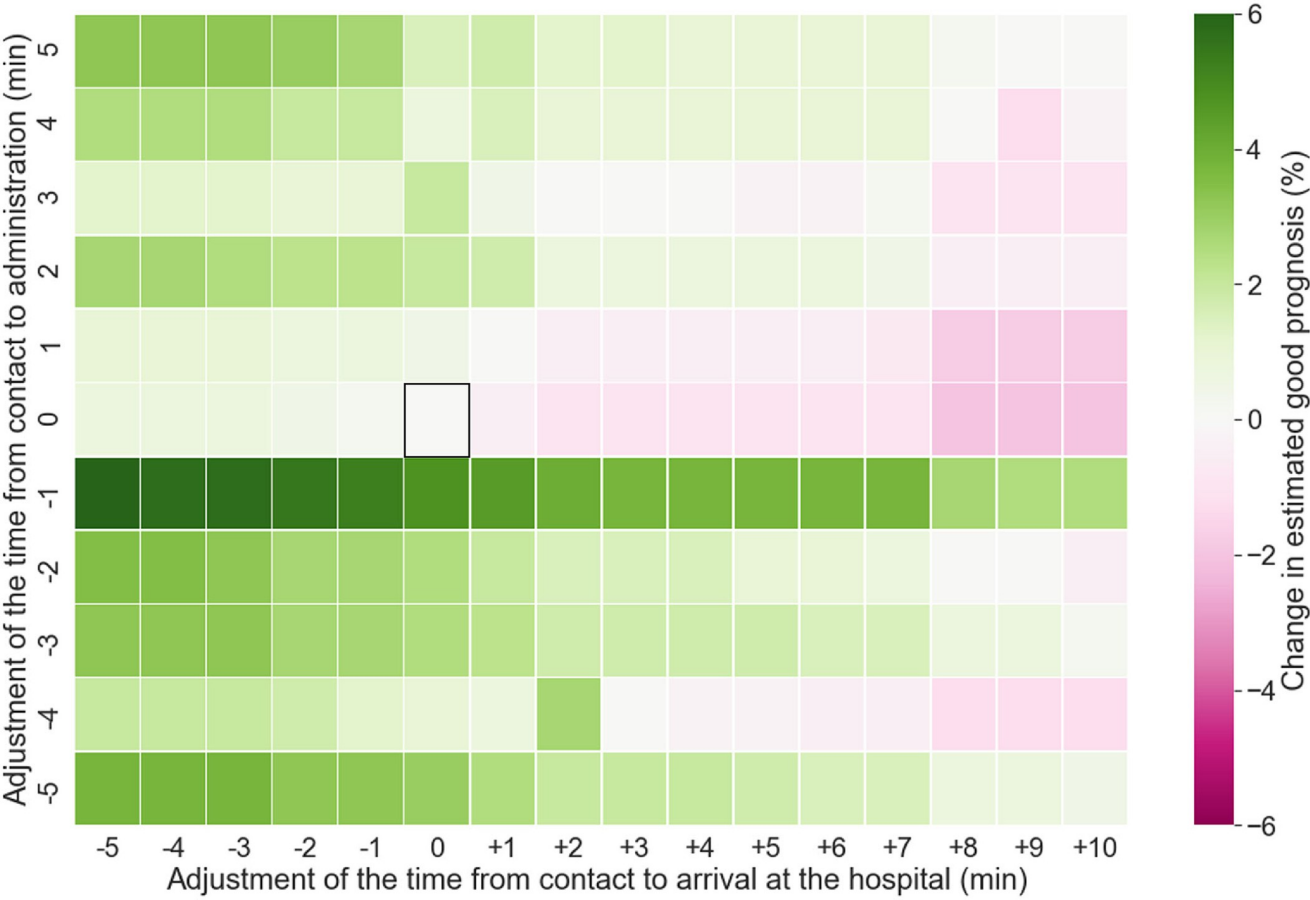
Modification of times from ambulance contact to hospital arrival and first-drug administration. The square box indicates no adjustments. The two ends of the color spectrum show a 6% increase and decrease in predicted CPC1/2, respectively. Overall, reducing the time for hospital arrival improved outcomes, whereas delays worsened them. Reducing the time to drug administration strengthened the effect of reducing the transport time, but reducing the transport time improved the prognosis even with delayed drug administration. CPC, cerebral performance category.

Figs 4 and 5 depict the effects of modifying the time parameters. The time elapsed between contact and arrival and the time to first defibrillation were adjusted. When the time of arrival at the hospital was exclusively adjusted, the prognosis was found to be better for shorter durations. Conversely, any delay in arrival at the hospital worsened the prognosis, as expected. Furthermore, a 1 min delay in defibrillation improved the prognosis. Overall, the combination of short transport and defibrillation durations improved prognosis, whereas any increase/decrease in the defibrillation time combined with prolonged transportation duration worsened the prognosis. Increases and decreases in defibrillation time of more than 3 min in a few training cases (S1 Fig) led to poor outcomes.

Next, we adjusted the times for hospital arrival and first-drug administration. Contrary to defibrillation, a 1 min reduction in the time for drug administration improved prognosis. Although the combination of short transportation duration and defibrillation time demonstrated the most improvement in prognosis, a short transportation duration improved prognosis even when drug administration was delayed. Conversely, shortening the drug-administration time also improved prognosis despite delays in hospital arrival. In both cases, an interaction between two factors was found to influence prognosis, instead of the monotonic effect of a single factor.

## Discussion

In this study, we developed a neurological prognostic model that demonstrates a full AUROC of 0.96 with weights adjusted to accurately predict the number of minority classes based on prehospital information. We simultaneously and consecutively adjusted the values of two EMS time factors in the model, thereby obtaining a graphical representation of non-monotonic, nonlinear effects on the predicted outcome. This is the first attempt to demonstrate that prognosis can be performed by considering the interaction between multiple EMS activity factors.

Prehospital EMS activities involve several considerations, including the choice of the intervention to be prioritized and whether they should be performed at the scene or prioritized for arrival at the hospital. Other considerations include whether a reduction in the time taken for each factor improves the outcome and whether an increase in time is acceptable. If yes, to what extent? Previous studies focused on single prognostic factors, such as early drug administration [25–29] or early defibrillation [24, 30]. However, because prognostic factors (e.g., defibrillation, drug administration, and transport times) are characterized by covariation and multicollinearity, the effects of the interaction between them must be considered. The possibility of predicting the interaction between these factors using a machine learning model has been previously reported [11], thereby increasing factor-effect representations in such models. However, most prognostic-factor-modification-effect studies focused on modifying the values of a single factor [12, 18]. Moreover, single-factor modification is insufficient for devising an EMS activities-improvement strategy. This study assessed the prognostic impact of simultaneously modifying two time factors that influence EMS activities.

The nonlinear prognostic impact of the two modifiers identified in this study could be used to prioritize EMS activities. Reducing transport time and time for interventions have both been reported to improve prognosis. However, performing all of these at the same time is difficult in the EMS setting; hence, a decision needs to be made as to whether transport or intervention should be prioritized. In this study, reducing the time before defibrillation improved outcomes. However, delays in transport time worsened the overall outcome; thus, avoiding delays in transport is the most important activity target. On the other hand, as regards drug administration, prolonging both transport time and time for drug administration has a similar impact on outcomes. Consequently, if one cannot be achieved earlier, priority should be given to the other to avoid a worsening outcome.

Previous resuscitation-improvement trials only accepted interventions with a positive effect based on the evidence obtained using statistical methods. In this regard, machine learning models theoretically allow for the modification of any parameter. Additionally, our study results confirm the prognostic impact of delayed interventions. An investigation of factors that worsen the outcome can provide the necessary information, particularly when normal activities are interrupted.

However, the prognostic impact after parameter modification should be interpreted cautiously. Only 21 (15%) patients for whom the parameters were modified in this study had a defibrillation time that was delayed by more than 3 min from contact. Because only a few instances of defibrillation times of more than 3 min were modified, the effect of time reduction may have been underestimated. By contrast, simulation is considered possible in the case of delay, particularly in transport time, to the extent that there may be actual examples in the training data. If the background data to be modified are not understood and the data are not modified to the extent that they are realistic, the results may be underestimated, risking misinterpretation. Therefore, prior to making any modifications to the population data, careful confirmation of the content is crucial, which can be facilitated by the proposed model.

### Limitations

This study has several limitations. First, the input data used to construct the prediction model were limited to 22 prehospital factors. Further, patient clinical/personal features were not included. Additionally, as regards factors prior to EMS arrival, we could not collect data on the impact of verbal instructions from the communication command centers on prognosis, and thus could not use them as a feature. Furthermore, cardiopulmonary resuscitation is evidently continued on arrival at the hospital; however, this was not evaluated in this study. Additionally, cardiac catheterization and target temperature management performed after hospitalization are known to affect the outcome [31]. Therefore, more accurate prediction models may be possible if therapeutic interventions, such as cardiac catheterization and targeted temperature management, are included as parameters for model construction [15]. However, we limited our research to prehospital information because we focused on EMS activities that are generally available in other areas. Furthermore, EMS activities that have not been collected cannot be evaluated in this model. For example, the depth and frequency of chest compressions are known to be important; however, we could not assess their effect. Second, the predictive accuracy of machine learning decreases in uncommon cases, which were included in the training model. Therefore, caution should be exercised when evaluating a small amount of data containing less well-trained cases. In this study, the prediction ability of the proposed model may have been reduced owing to the consideration of a wide range of time reduction or extension. Therefore, only a few cases were available for training the machine learning model. However, because the effect of modification on the predictive ability could not be evaluated, we could not determine the modification threshold of the predictive potential. Therefore, the preferred use of the results is to estimate the direction in which outcome is improved by two modified factors, rather than to focus on cases with large adjustments. This problem can be minimized by using a training dataset with a large number of intervention time cases, of which there were only a few in this study. Third, the findings of this study cannot be adapted to other regions because adaptation is limited to the region where the training data were collected. We consider that the machine learning model presented in this study can be validated for external data if the dataset is based on the Utstein style. However, the potential existence of unknown interactions between factors that were not identified in this study cannot be ruled out, in which case the hyperparameters of the model may need to be modified. In addition, the findings of machine learning models are affected by several biases inherent in the training data, thereby resulting in inaccurate decision-making [32]. Therefore, the construction of a prognostic model that considers the factor-interaction results obtained using the proposed method must be validated using data collected from other regions. Finally, this study used only prehospital information in the Utstein style. The same method may be used in regions with such data available; however, external validation is required to verify whether the best prediction model can be reproduced in other regions. Furthermore, any change in resuscitation procedures that are not currently used, for example, the introduction of new medications or equipment, will require the introduction of additional factors into this model.

## Conclusions

A machine learning prognostic model trained on prehospital data allowed us to evaluate the interaction effect of two simultaneously modified parameters on outcome. The impact of each parameter modification on outcome may provide useful information for developing measures to improve EMS activities.

## Supporting information

S1 Fig Distribution of time factors used for adjustment. EMS, emergency medical services.
(TIF)

S2 Fig Sensitivity, specificity, diagnostic accuracy, and AUC for each of the fourfold cross-validation models and ROC curves. AUC: area under curve; CI: confidence interval.
(TIF)

S1 Table Prehospital emergency medical services activity records data and outcomes used in this study. (XLSX)

S2 Table Patient background characteristics. (XLSX)
