# Supplementary figures and images for: Visual assessment of interactions among resuscitation activity factors in out-of-hospital cardiopulmonary arrest using a machine learning model
Source: PLoS One. 2022 Sep 6;17(9):e0273787. doi: 10.1371/journal.pone.0273787 (PMC9447882; doi:10.1371/journal.pone.0273787)

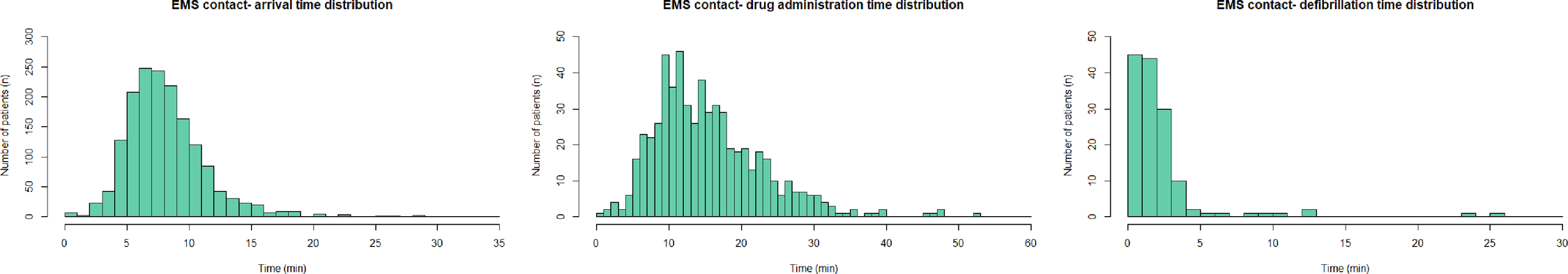

Supplement: S1 Fig — EMS, emergency medical services. (TIF) [file pone.0273787.s001.tif]

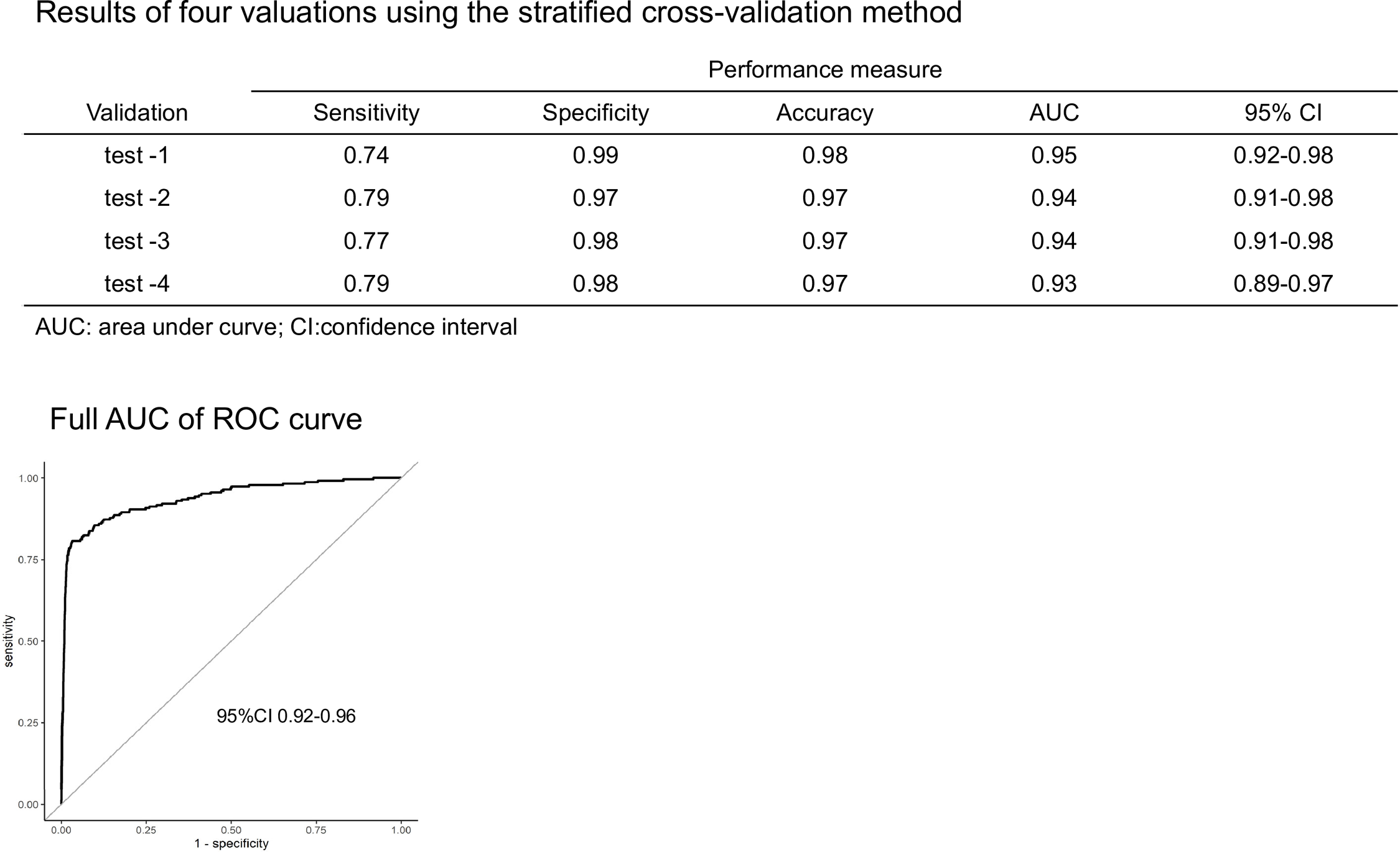

Supplement: S2 Fig — AUC: area under curve; CI: confidence interval. (TIF) [file pone.0273787.s002.tif]
